# Structural and Energetic Insights into SARS-CoV-2 Evolution: Analysis of hACE2–RBD Binding in Wild-Type, Delta, and Omicron Subvariants

**DOI:** 10.3390/ijms26083776

**Published:** 2025-04-17

**Authors:** Can Tang, Cecylia S. Lupala, Ding Wang, Xiangcheng Li, Lei-Han Tang, Xuefei Li

**Affiliations:** 1 State Key Laboratory of Quantitative Synthetic Biology, Shenzhen Institute of Synthetic Biology, Shenzhen Institutes of Advanced Technology, Chinese Academy of Sciences, Shenzhen 518055, China; c.tang@siat.ac.cn; 2 University of Chinese Academy of Sciences, Beijing 100049, China; 3 Department of Physics, Hong Kong Baptist University, Hong Kong SAR, China; 19481705@life.hkbu.edu.hk; 4 School of Life Science and Technology, ShanghaiTech University, Shanghai 201210, China; lixch2023@shanghaitech.edu.cn; 5 Shanghai Institute for Advanced Immunochemical Studies, ShanghaiTech University, Shanghai 201210, China; 6 Center for Interdisciplinary Studies, Westlake University, Hangzhou 310024, China; tangleihan@westlake.edu.cn

**Keywords:** SARS-CoV-2, RBD-variants, molecular dynamics, binding strength

## Abstract

The evolution of SARS-CoV-2, particularly the emergence of Omicron variants, has raised questions regarding changes in its binding affinity to the human angiotensin-converting enzyme 2 receptor (hACE2). Understanding the impact of mutations on the interaction between the receptor-binding domain (RBD) of the spike protein and hACE2 is critical for evaluating viral transmissibility, immune evasion, and the efficacy of therapeutic strategies. Here, we used molecular dynamics (MD) simulations and binding energy calculations to investigate the structural and energetic differences between the hACE2- RBD complexes of wild-type (WT), Delta, and Omicron subvariants. Our results indicate that the Delta and the first Omicron variants showed the highest and the second-highest binding energy among the variants studied. Furthermore, while Omicron variants exhibit increased structural stability and altered electrostatic potential at the hACE2–RBD interface when compared to the ancestral WT, their binding strength to hACE2 does not consistently increase with viral evolution. Moreover, newer Omicron subvariants like JN.1 exhibit a bimodal conformational strategy, alternating between a high-affinity state for hACE2 and a low-affinity state, which could potentially facilitate immune evasion. These findings suggest that, in addition to enhanced hACE2 binding affinity, other factors, such as immune evasion and structural adaptability, shape SARS-CoV-2 evolution.

## 1. Introduction

The evolution of SARS-CoV-2 has led to the emergence of multiple variants of concern (VOCs), with the Omicron lineage (B.1.1.529) standing out due to its extensive mutational profile in the RBD of the spike protein. These mutations have profound implications for viral infectivity, immune evasion, and vaccine efficacy, which have become central concerns in managing the COVID-19 pandemic. Since its identification in late 2021, Omicron and its subvariants have rapidly spread globally, driven by enhanced transmissibility and the potential for immune escape [1,2,3].

The spike protein of SARS-CoV-2 plays a key role in mediating viral entry by binding to the hACE2 [4,5]. Mutations within the spike protein, especially in the RBD, can significantly alter the binding affinity to hACE2, thereby influencing viral infectivity and transmission dynamics [6]. Key mutations found in the Omicron lineage such as K417N, E484K, Q493R, and N501Y have raised concerns about increased contagiousness and reduced vaccine efficacy [7,8]. Furthermore, some of the variants have been shown to exhibit reduced neutralization by antibodies elicited through vaccination or previous infection, increasing the likelihood of breakthrough infections and reinfections in certain populations [9].

The relationship between binding affinity and viral transmissibility remains a central focus of research in understanding the mechanisms driving SARS-CoV-2 evolution [6]. Early studies indicated that Omicron subvariants exhibit binding strength to hACE2 similar to that of the WT virus (i.e., the ancestral SARS-CoV-2 reference strain first identified in late 2019 [10]) and the previously dominant Delta variant [11]. However, subsequent research has raised questions about this relationship, suggesting that despite an increased number of mutations, the overall binding affinity of the Omicron RBD to hACE2 might be weaker than that of Delta, prompting questions regarding the role of binding affinity in viral transmissibility and evolution [12]. Notably, specific mutations in Omicron subvariants, such as N440K, Q493R, and Q498R, have been shown to enhance electrostatic interactions with hACE2, which could theoretically improve binding affinity. Conversely, other mutations appear to weaken these interactions, highlighting the complexity of assessing their collective impact on viral infectivity and immune evasion [7]. Further insights into the Omicron variant’s evolutionary adaptations from MD studies reveal that mutations in early Omicron sub-lineages (BA.1–BA.4) progressively enhanced RBD–hACE2 interactions by optimizing allosteric communication pathways. These adaptations enhanced binding efficiency and structural flexibility, potentially contributing to increased transmissibility and antibody escape [13]. This nuanced interplay of mutations underscores the need for further structural and biophysical studies to understand the determinants of viral transmissibility and immune escape.

The phylogenetic origins of Omicron remain uncertain, with no direct lineage traced to previous VOCs such as Alpha, Beta, Delta, or Gamma [14,15]. The evolution of SARS-CoV-2 raises important questions about the mechanisms driving viral adaptation and the selective pressures on its spike protein. Early reports speculated that the rapid emergence of Omicrons might reflect viral adaptations in response to immune pressures or changes in host population dynamics. The origin and evolution of the Omicron variant have been attributed to several hypotheses, including its circulation in under-surveilled populations, emergence in a chronically infected individual, or zoonotic spillover from a nonhuman species [16,17]. Additionally, the potential for the evolution of Omicron to prioritize immune evasion rather than enhancing receptor binding affinity has been suggested, as studies have shown that certain mutations may optimize the ability of the virus to evade neutralizing antibodies rather than strengthen its interaction with hACE2 [18,19].

**Figure 1:**
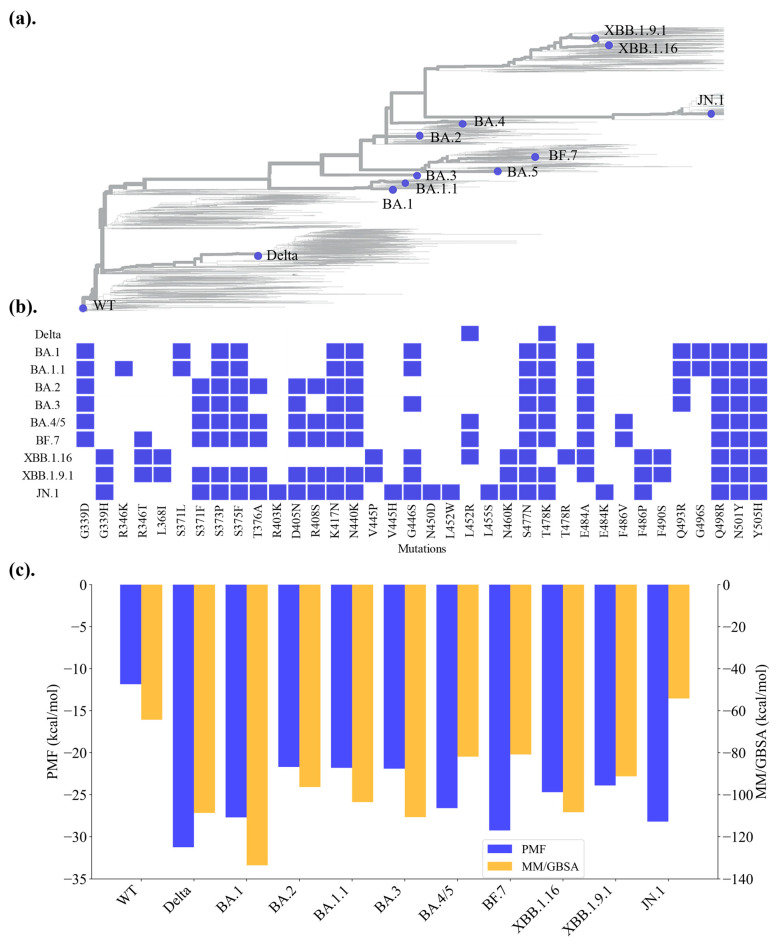
**The evolution and hACE2–RBD binding energies of SARS-CoV-2 variants.** (**a**) Phylogenetic relationship of SARS-CoV-2 variants. Adapted from Nextclade accessed on 1 January 2024 (https://clades.nextstrain.org/). The variants of interest in this work are labeled. (**b**) Alignment of RBD mutation sites of SARS-CoV-2 variants. The mutation sites are highlighted. (**c**) Comparison of binding energies between the RBD and hACE2 receptor for different SARS-CoV-2 variants, showing PMF values (blue) averaged from two independent trajectories and MM/GBSA values (orange) calculated for the most sampled conformation in the same trajectories.

Omicron comprises three primary sub-lineages—BA.1, BA.2, and BA.3—with BA.4 and BA.5 emerging as derivatives of BA.2 and sharing an identical RBD sequence. BF.7, an abbreviation of BA.5.2.1.7, represents a third-generation subvariant of Omicron BA.5 [20]. The recently dominant strain, JN.1, is also a descendant of BA.2 [21]. Additionally, the XBB lineage is a recombinant strain formed from the genomes of two distinct SARS-CoV-2 strains, both originating from the Omicron variant BA.2 [22]. XBB.1.16, commonly referred to as Arcturus, was first identified in India, and is a hybrid variant resulting from the recombination of Omicron subvariants BA.2.10.1 and BA.2.75 (Figure 1a,b). To understand the molecular basis of these evolutionary changes, it is essential to investigate the structural and functional characteristics within these sub-lineages. A comprehensive analysis of the interactions between the RBD and the hACE2 receptor across Omicron’s evolutionary trajectory is pivotal in unraveling the mechanisms underlying viral transmissibility and immune evasion.

In this work, we used MD simulations to refine the structural complexes and study the interactions between the hACE2 receptor and the RBDs of ten different Omicron variants (BA.1, BA.1.1, BA.2, BA.3, BA.4/BA.5, BF.7, XBB.1.16, XBB.1.9.1, and JN.1). We compared these variants to the WT and Delta variants to elucidate differences in molecular structure, dynamics, and binding affinities. The Delta variant was selected due to its high transmissibility and strong receptor–binding affinity, making it a key comparator for later variants. In contrast, earlier variants such as Alpha and Beta were excluded, as their interactions with hACE2 have been extensively characterized in previous studies [23,24,25], and their impact was superseded by Omicron, which exhibits greater structural and functional divergence [26,27].

Previous studies have employed MD simulations to investigate SARS-CoV-2 RBD-hACE2 binding properties [7,11,23]. However, most focused on a limited number of variants or relied on a single computational approach. In contrast, our study traces the evolutionary trajectory of SARS-CoV-2, analyzing the progression from the WT strain to the pre-Omicron dominant Delta variant, followed by ten chronologically emerging Omicron subvariants. The inclusion of early lineages (BA.1, BA.1.1, BA.2, BA.3), intermediate descendants (BA.4/5, BF.7), and recent variants (XBB.1.16.16, XBB.1.9.1 [early 2023], and JN.1 [late 2023]) enables a comprehensive comparative analysis of molecular structure, dynamics, and binding affinities across multiple evolutionary stages. Furthermore, our study integrates multiple computational methodologies to provide a more robust and holistic assessment of hACE2–RBD interactions. We combine equilibrium MD simulations, Molecular Mechanics/Generalized Born Surface Area (MM/GBSA) calculations, and Potential of Mean Force (PMF) analysis to capture both binding energetics and free energy landscapes. This multi-faceted approach enhances our understanding of how SARS-CoV-2 evolution influences receptor binding and immune evasion strategies, offering novel insights beyond previous computational studies.

Our results reveal that mutations in the RBD significantly influence its interaction with hACE2. Compared to WT, Omicron variants exhibit a more stable RBD structure and maintain robust interactions with hACE2. However, among the Omicron sub-lineages, binding energies do not always increase for newly emerged variants. Interestingly, the binding energies for Omicron variants were generally lower than those of Delta variants. These findings suggest that structural adaptations do not necessarily correlate with stronger binding to hACE2. Notably, we discovered that BA.3 and JN.1 exhibit dual conformational subpopulations, which suggest a potential balance between structural rigidity and functional flexibility. This duality enables strong hACE2 binding while facilitating immune evasion by regulating RBD conformations, highlighting an evolutionary trade-off between receptor affinity and immune evasion. A previous study suggested that JN.1 escapes antibody binding through steric hindrance while maintaining hACE2 binding through compensatory interactions [28]. These results underscore the interplay between receptor binding affinity, immune evasion, and structural stability, suggesting the evolutionary balance SARS-CoV-2 maintains between receptor binding and avoiding host immune responses [26].

## 2. Results and Discussion

### 2.1. Model Assessment and Validation

For the modeled structures of BA.4/BA.5, XBB.1.9.1, XBB.1.16, BF.7, and JN.1, 96–99% of the amino acid residues are located within the energetically favored regions of the Ramachandran plots [29] (Figure S1), indicating good stereochemical quality. MolProbity evaluation further confirms the reliability of these predicted structures, as all exhibited acceptable quality metrics. A lower MolProbity score is indicative of better structural quality at a given resolution [30].

For comparison, the hACE2-WT_RBD complex, resolved at a resolution of 2.50 Å (PDB ID:6M0J), had a MolProbity score of 1.10, which served as the template for modeling. The predicted RBD structures of BA.4/BA.5, XBB.1.9.1, XBB.1.16, BF.7, and JN.1 have MolProbity scores of 1.43, 1.62, 1.55, 1.43, and 0.83, respectively. These scores reflect structural quality comparable to or better than the template, underscoring the reliability of the modeling process.

### 2.2. Binding Energies Reveal No Consistent Increase in Affinity to hACE2 with Evolution

To evaluate the binding affinities of SARS-CoV-2 RBD variants with hACE2, both the MM/GBSA method and PMF approach were used. MM/GBSA estimates binding energy by considering energy decomposition and solvation effects, while PMF captures free energy changes along specific reaction coordinates. Our results show that compared to the ancestral WT strain, Omicron variants show higher binding affinities (Figure 1c). However, the Delta strain shows the highest binding affinity among all strains studied. This is in agreement with previous studies reporting that Omicron variants generally exhibit lower binding affinity to hACE2 compared to the Delta variant [12,23,31]. Specifically, variants such as Delta and BA.1 consistently exhibit robust binding affinities across both methods, with MM/GBSA scores of −107.3 kcal/mol (Delta) and −133.5 kcal/mol (BA.1), and PMF values of −31.3 kcal/mol (Delta) and −27.7 kcal/mol (BA.1), and they were classified as strong binders. Conversely, the ancestral WT showed a low PMF value and less favorable MM/GBSA score of −11.9 kcal/mol and −64.3 kcal/mol, indicating significantly weaker binding interactions. PMF values indicate that BA.1 shows a binding energy 2.5 times stronger than the WT, consistent with previous findings [32,33].

The variants can be grouped into strong, moderate and weak binders according to the calculated binding affinities (Figure 1c, Table S1). Strong binders, such as Delta and BA.1, exhibited consistently high binding affinities across both methods, while weaker binding was observed in the ancestral WT. XBB.1.16 and BA.3 show relatively higher MM/GBSA scores (−108.3 and −110.7 kcal/mol, respectively) indicative of stronger binding affinities compared to their moderate PMF values (−24.7 and −21.9 kcal/mol, respectively). PMF calculations directly probe the detailed energy landscapes and entropic contributions along reaction coordinates, but the computed PMF values are significantly affected by factors such as initial conformations, input trajectories, the chosen reaction coordinate, and the sampling of conformational space within each umbrella sampling (US) window [34]. In contrast, MM/GBSA estimates the binding energy that accounts for solvation and decomposition effects but may overlook conformational or entropic contributions captured using the PMF approach [35]. The agreement between the two methods in identifying strong and weak binders strengthens confidence in these classifications. However, the variability observed among moderate binders suggests that additional analyses may be necessary to refine their classification.

Comparing the binding affinities among the Omicron variants reveals that binding strength has not consistently increased throughout viral evolution. For example, early Omicron variants such as BA.1 demonstrate relatively strong binding affinities, as indicated by MM/GBSA scores and PMF calculations. In contrast, later variants like XBB.1.16 and XBB.1.9.1 exhibit reduced binding strengths compared with BA.1. This apparent decline in binding affinity among later Omicron variants aligns with findings from other studies reporting inconsistent binding affinities across Omicron sub-lineages [36]. These results suggest that the evolution of SARS-CoV-2 is not driven solely by enhanced binding affinity to hACE2, but is also influenced by other factors. To gain a more comprehensive understanding of evolutionary trends, we proceeded to conduct a detailed analysis of the variants.

### 2.3. Inconsistently Enhanced Electrostatic Potential and Binding Interactions in Omicron Variants

**Figure 2:**
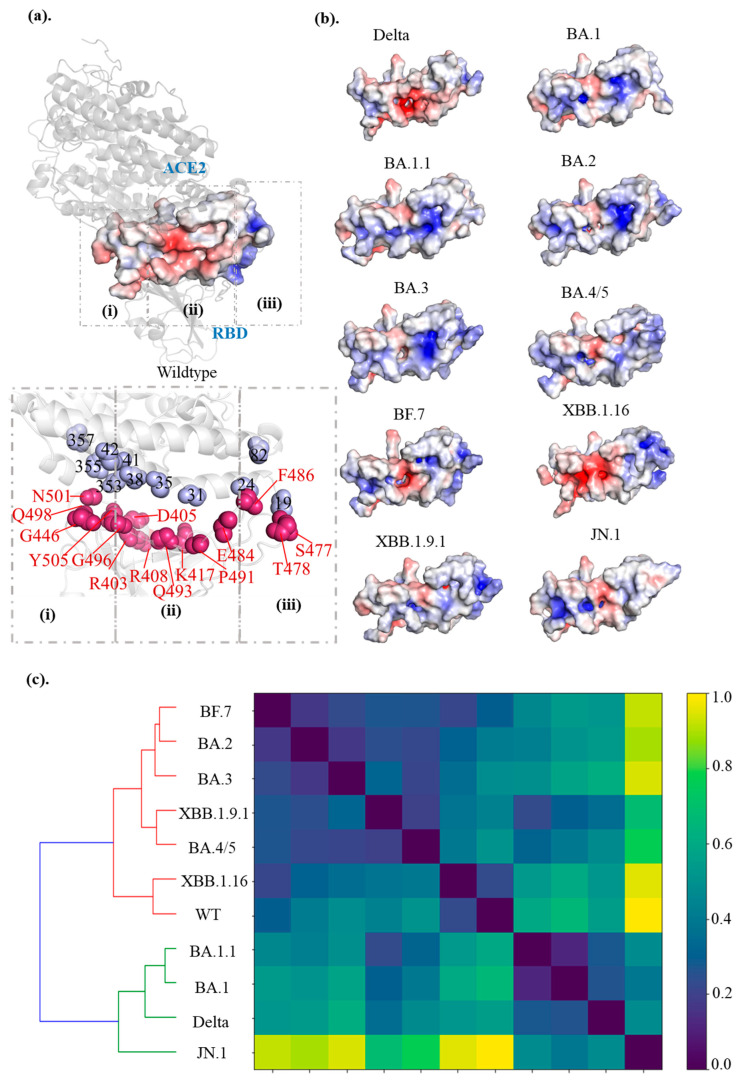
**Electrostatic potential distribution at the hACE2–RBD interface.** (**a**) Schematic of the hACE2–RBD binding interface showing positive and negative charge distribution for the WT SARS-CoV-2. (**b**) Electrostatic potentials map of the hACE2–RBD interface for 10 SARS-CoV-2 variants, with a gradient ranging from −5 kBT/e (red, negative potential) to +5 kBT/e (blue, positive potential). (**c**) Hierarchical clustering of electrostatic surfaces maps, revealing the electrostatic diversity and evolutionary relationships among the variants. The color bar indicates the distance between pair-wise variants.

Previous MD simulation studies have highlighted enhanced electrostatic interactions as key contributors to the strong binding affinity observed in Omicron variants [36,37,38]. To explore this further, we calculated the electrostatic potentials by solving the Poisson–Boltzmann equation for the interface residues between hACE2 and variants RBD [39]. The hACE2–RBD interface was divided into three “patches” for analysis. In the hACE2–WT_RBD complex, patch (i), which encompasses residues around N501, exhibits a mix of different charged residues that are relatively low in potential and closely dispersed. In contrast, patch (ii) centers on the middle interface, and patch (iii) near the RBD loop shows significantly higher concentrations of negative and positive electrostatic potential residues, respectively (Figure 2a).

Our results show that electrostatic potential increased from WT to early Omicron subvariants. However, later Omicron subvariants exhibited a shift in electrostatic potential mapping, reflected through changes in the spatial distribution of charged residues at the hACE2–RBD interface (Figure 2b and Figure S2). In early Omicron subvariants, patch (i) mutations, such as Q498R, N501Y and Y505H, enhance the positive charge, complementing the negatively charged residues on hACE2 (e.g., D38, Y41, Q42, and D355). Substitutions in patches (ii) and (iii), including R403K, K484E, R493Q, and T478K, induce highly charged residues, further strengthening electrostatic interactions with the negatively charged hACE2 binding surface. However, the evolution of Omicron variants did not exhibit a consistent increase in electrostatic potential. Recombinant variants like XBBs, which combine mutations from different lineages, change the spatial arrangement and electrostatic balance of the hACE2–RBD interface. For example, the electrostatic surface distribution of XBB.1.9.1 and XBB.1.16 closely resembles that of the WT, maintaining a relatively balanced interface. In contrast, JN.1 displays a reversed electrostatic surface distribution compared to the WT (Figure 2b,c), suggesting a marked shift in the potential landscape, which could impact receptor recognition and binding dynamics. This cumulative effect of mutations reshapes the interface potential, influencing the binding affinity between hACE2 and RBDs [40].

**Figure 3:**
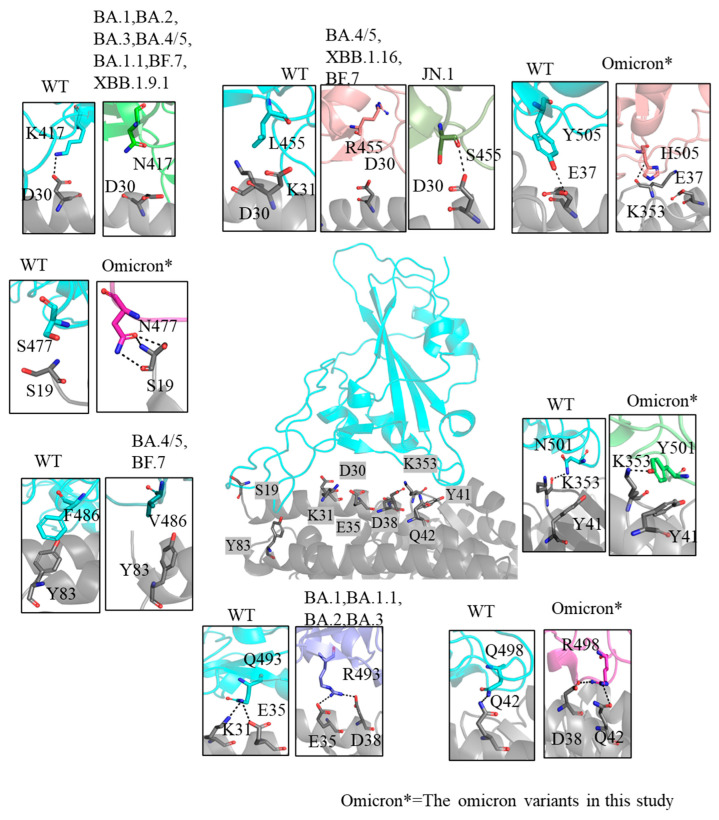
**Interaction details for key residues at the binding interface.** The grey region corresponds to hACE2. The cyan regions represent the WT RBD while the remaining colors show Omicron subvariant RBDs. Key interacting residues are highlighted and labeled to illustrate how mutations in these positions modify interactions with hACE2. Black dashed lines indicate hydrogen bonds, demonstrating their role in stabilizing the interface. Mutations alter hydrogen bond patterns, leading to diverse changes in binding strength and interaction dynamics.

Beyond electrostatic potential, these mutations also impact other interactions critical to binding stability. For instance, N501Y and Y505H mutations enhance binding through additional interactions, such as a π–π interaction with Y41 and a hydrogen bond with K353, respectively, further stabilizing the interface in patch (i) (Figure 3). To explore these interactions further, we conducted two replicate 500 ns equilibrium MD simulations and quantified interaction strength variations using data from the stable final 100 ns of each simulation. Hydrogen bonds (H-bonds) are key contributors to the stabilization of protein–protein complexes, significantly reinforcing the structural integrity of the interface [41]. BA.1 demonstrated the highest number of H-bonds (9.99) among the analyzed variants, suggesting its strong affinity for hACE2 (Table 1). Besides this, the Omicron mutations enhance H-bond stability. For example, the Y501 residue in the Omicron RBD consistently forms a stable H-bond with K353 on hACE2 (Figure S3), and the Q493R mutation exhibits higher H-bond occupancy at position 493 compared to the WT, thereby strengthening local binding interactions [42]. Beyond H-bonds, an increased number of atomic contacts and a larger buried surface area at the interface further stabilize the hACE2–RBD complex, indicating stronger binding affinity. Delta exhibited the highest number of atomic contacts (316.28) and a substantial buried surface area of 18.42 nm^2^, reflecting its stronger interaction with hACE2 relative to the WT. The statistics of buried surface areas align with the number of observed contacts—most Omicron variants, particularly JN.1 and BA.3, have larger buried surface areas and more contacts than WT (Table 1).

**Figure 4:**
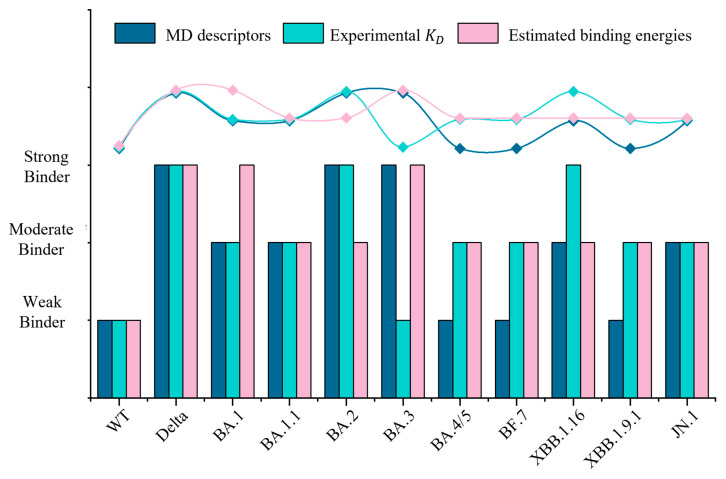
**Computational and experimental evaluation of RBD-hACE2 binding affinities.** The bar chart represents binding strengths as determined by MD-derived descriptors, experimental *K*_D_, and theoretically estimated binding energies. Binding trends, represented by curves, indicate consistency across methods, with no significant increase in binding affinity observed during variants evolution.

Our analysis of interactions aligns with our results on binding affinities, where a higher number of H-bonds, more atomic contacts, and larger buried surface areas characterize higher binding affinities and, thus, strong binders. For instance, BA.3 demonstrates a high H-bond count and the highest buried surface area, justifying the classification as a strong binder. In contrast, weaker binders like XBB.1.9.1 and BA.4/5 exhibit significantly fewer H-bonds and smaller buried surface areas, indicative of less stable interactions. This classification is further validated by experimental dissociation equilibrium constant (*K*_D_) values (Table 1 and Tables S2–S4). For instance, Delta exhibits *K*_D_ values in the nanomolar range [43,44], consistent with a strong binding affinity. Most Omicron subvariants exhibit lower *K*_D_ values than the WT variant. However, *K*_D_ values fluctuate among Omicron subvariants, as mutations that facilitate immune evasion by altering RBD epitopes may also subtly affect hACE2 binding. Notably, while our MD analysis aligns with this classification for BA.1 and XBB.1.9.1 as moderate or weak binders, MM/GBSA contradicts with this result (Figure 4). MM/GBSA overlooks conformational or entropic contributions, making it important to study the structural stability and flexibility that accounts for these contributions [50].

To summarize the computational and experimental findings, the binding affinity of Omicron variants for hACE2 did not increase uniformly throughout viral evolution (Figure 4). Early Omicron variants (e.g., BA.1) exhibit strong binding affinity, while later variants (e.g., XBB.1.9.1) show a decreased binding affinity compared to earlier strains like BA.1 and BA.2. Importantly, our computational results align closely with experimental trends—the Delta variant binds to hACE2 significantly more strongly than the WT variant, whereas Omicron subvariants display variable binding affinities. However, discrepancies between computational and experimental outcomes occasionally arise due to limitations in simulation methods, such as restricted timescales and insufficient conformational sampling [51], as well as experimental factors. For instance, in surface plasmon resonance (SPR) assays, protein immobilization on sensor chips can alter ligand conformations, potentially introducing systematic bias [52].

The resulting inconsistency in binding affinity enhancement suggests a trade-off between receptor binding and other evolutionary pressures, such as immune evasion and structural stability. For example, mutations in Omicron subvariants not only alter binding affinity but also modify the RBD’s antigenic surface, thereby enhancing immune escape [48,49]. These suggest that evolution might not be driven by enhancing hACE2 binding affinity only. A more comprehensive analysis is needed to fully understand the evolutionary trends of SARS-CoV-2.

### 2.4. Enhanced Global Stability and Diverse Local Flexibility in Omicron Variants

**Figure 5:**
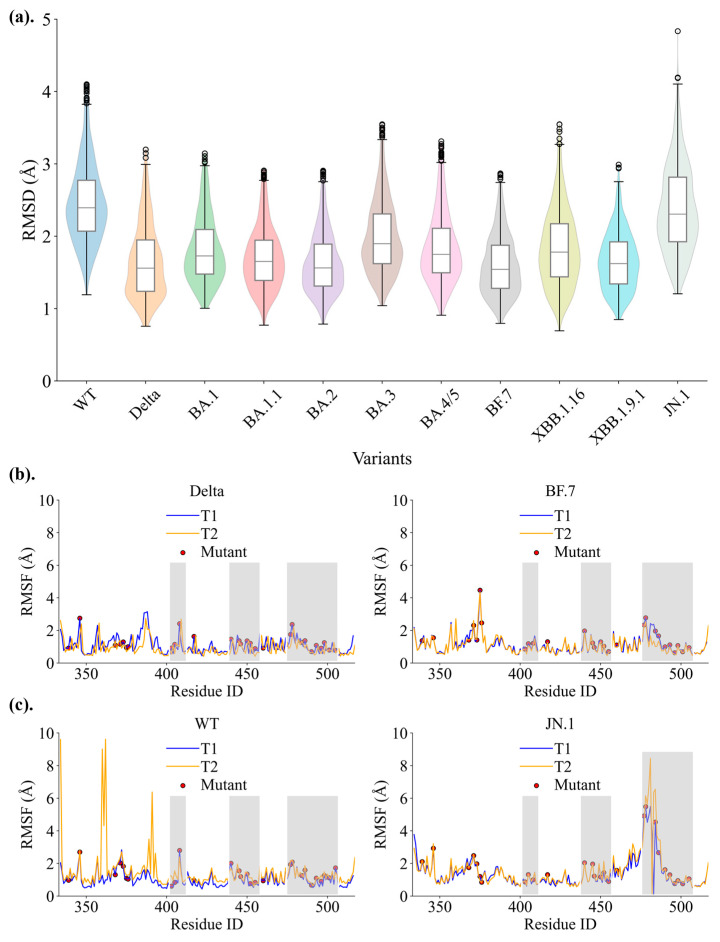
**Stability of hACE2–RBD Complexes.** (**a**) The RMSD of the backbone atoms of the RBD with respect to the starting structure for two trajectories T1 and T2, with outliers indicated by open circles. (**b**) Per-residue fluctuation (RMSF) for stable variants such Delta and BF.7 shows minimal fluctuations, while (**c**) RMSF profiles of WT and JN.1 show elevated per-residue fluctuations. Mutation residues are labeled, and the interfaces, referring to the contact regions of the RBD that are in direct contact with hACE2, are highlighted with grey shadow.

To evaluate the structural stability of SARS-CoV-2 variants, Root Mean Square Deviations (RMSD) along the chain were analyzed across MD trajectories, with higher RMSD values indicating greater conformational instability. The WT RBD displays the highest median RMSD (~2.4 Å), whereas Omicron variants consistently exhibit reduced RMSD values (<2.3 Å), reflecting enhanced structural stability (Figure 5a). The complex RMSD values also show that the Omicron variants are more stable than WT (Figure S4). Root Mean Square Fluctuation (RMSF) data further support these observations (Figure 5b,c and Figure S5). Omicron variants consistently display lower per-residue fluctuations in key interface regions compared to the WT, indicative of reduced flexibility (Figure 5b). In contrast, JN.1 exhibits higher RMSF values compared to other Omicron variants, particularly at the loops’ tip residues (residues 470–490), reflecting increased conformational adaptability (Figure 5c).

These findings suggest that Omicron variants generally exhibit reduced RBD flexibility compared to WT, which correlates with enhanced binding stability [27]. However, this increased rigidity may come at the cost of reduced dynamic adaptability during interactions, potentially affecting the ability to respond to diverse receptor conformations or environmental changes. Supporting this, previous studies have shown that interactions involving mutant residues at the loop such as T478K and S477N also contribute significantly. For instance, T478K enhances binding by stabilizing nearby polar interactions, while S477N increases receptor-binding motif flexibility, affecting distal binding dynamics [40,53]. The RMSF data also highlight differences in flexibility among variants. While Delta variants optimize static binding strength at the expense of dynamic adaptability, JN.1 leverages greater flexibility to maintain versatile interactions. These findings suggest a complex interplay between stability and binding adaptability in the evolution of SARS-CoV-2 variants.

**Figure 6:**
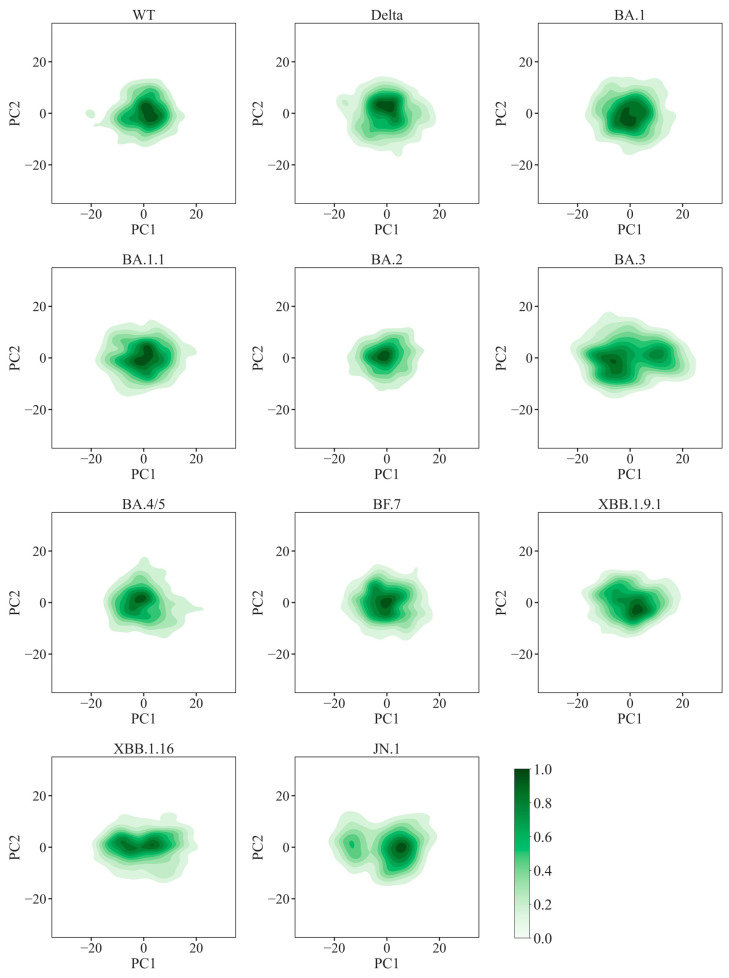
**The projection of the trajectories of SARS-COV-2 RBD along the principal components PC1 and PC2.** The density contours indicate the frequency of the sampled conformations. Most variants exhibit a singular conformational region, while BA.3, XBB.1.16 and JN.1 highlight distinct conformational subpopulations.

To better understand the structural adaptability of variants, Principal Component Analysis (PCA) was employed using trajectories from two independent simulations to capture dynamic motions, with PC1 representing the primary variance in RBD conformational dynamics. Narrower distributions in the PCA space correspond to restricted motions and enhanced structural stability. The WT RBD exhibited intermediate stability, whereas Delta and certain Omicron variants (e.g., BA.2, BF.7) displayed compact PC1 distributions, indicative of structural rigidity. Conversely, BA.3 and JN.1 exhibited broader PC1 distributions and bimodal clustering, suggesting distinct conformational subpopulations (Figure 6 and Figure S6). Eigenvalue analyses further corroborated these observations; the PC1 eigenvalues (103.11 and 90.15) and variance ratios (0.35 and 0.33) for BA.3 and JN.1 were the highest, indicating enhanced stability and dynamic adaptability. In contrast, BA.2 and BF.7 showed lower PC1 eigenvalues (47.35 and 53.46) and variance ratios (0.19 and 0.20), reflecting increased rigidity (Table S5).

Moreover, structural comparisons of the multiple conformations from BA.3 and JN.1 show differences mostly on the loop at the tip of RBD and patch (iii) of the interface (Figure S7). Despite these localized fluctuations, most variants retained the critical “hook-like” architecture at the interface essential for hACE2 binding (Figure S8), which is characterized by a stable and well-ordered conformation of the receptor binding motif (RBM) tip [54]. In contrast, variants with increased RBM mobility, such as JN.1, exhibited a transition from the “hook” conformation to a more dynamic state (Figure S7), which has been suggested to enable greater flexibility at the loop region in variants with high fluctuation [55].

Free Energy Landscape (FEL) analysis is valuable for uncovering the energy-related mechanisms underlying protein conformational shifts [56]. In our analysis, variants like WT, Delta, and BF.7 show centralized, symmetrical basins indicative of stable conformations (Figure S9). In contrast, BA.3 and JN.1 show broader and fragmented landscapes, signifying increased flexibility. The two variants display a unique bimodal conformation distribution with multiple deep basins, highlighting distinct structural modes. The dual conformations in “bimodal subvariants” may reflect an evolutionary advantage to the variant, such as optimizing receptor binding in one conformation and immune escape in another, as we discuss in more detail below.

Overall, our simulations show that Omicron variants are generally more structurally stable and rigid than the WT strain. However, BA.3 and JN.1 flexibility may offer other advantages.

### 2.5. Insights into Immune Evasion Features

**Figure 7:**
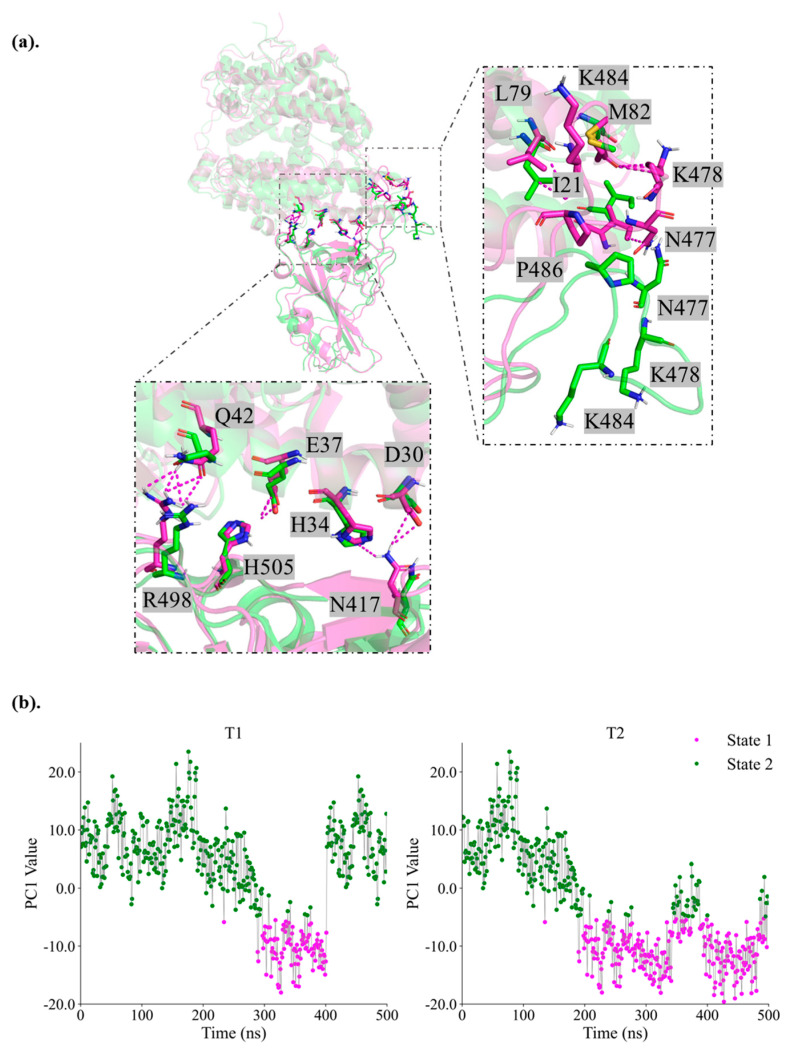
**Structural conformations of the JN.1 RBD-hACE2 complex derived from MD simulations.** (**a**) Two distinct binding conformations are shown: a high-affinity state (−109.2 11 kcal/mol, magenta) and the most sampled, a low-affinity state (−54.19 kcal/mol, green). The zoomed-in view highlights key RBD residues involved in hACE2 binding, many of which are targeted by neutralizing antibodies, emphasizing the functional significance of these conformations. The dashed lines delineate the contacts between RBD residues and hACE2 residues. (**b**) The PC1 values JN.1 in two independent simulations, showing the switches between the two states.

The enhanced flexibility observed in variants BA.3 and JN.1, as evidenced by RMSF, PCA and FEL analysis, may facilitate the conformational masking of neutralizing epitopes, further contributing to immune escape [57]. Specifically, BA.3 adopts two conformations with strong binding strengths to hACE2 (−112.1 kcal/mol and −120 kcal/mol), indicative of optimized receptor binding without a significant trade-off for immune evasion. Consistently, virus neutralization assays suggest that BA.3 is not a major immune-escape variant [58]. In contrast, JN.1 demonstrates a dynamic bimodal conformational strategy, which features switching between two distinct states. These states, identified through PCA and FEL mapping, exhibit differential binding affinities with calculated energies of −54.19 kcal/mol for the weaker state and −109.2 kcal/mol for the stronger state (Figure 7). The weaker conformation is characterized by reduced interactions with hACE2 at key RBD residues (e.g., N477, K478, P486, N417, R498, H505) known to influence the antibodies binding (Figure 7a) [59,60,61].

Such a bimodal conformational strategy aligns with previous studies on the full-length spike protein, which hypothesized that SARS-CoV-2 balances infection potency and immune evasion by regulating spike protein conformations [62]. As the early variant, BA.3 shows strong binding strength in two conformations, which may be due to its preference for maintaining infection efficiency. The bimodal conformation strategy of JN.1 shows a more complex adaptive mechanism by balancing immune escape and infectivity in different conformations. Such structural adaptations underscore a trade-off strategy whereby the virus maintains sufficient binding to hACE2 while evading host immune responses, a feature that could explain the relatively moderate binding affinities in some Omicron sub-lineages despite their extensive transmissibility. Studies on key mutations also support this balancing of receptor engagement and immune evasion [48].

The bimodal conformation strategy is an advanced evolutionary adaptation that dynamically balances receptor binding and immune evasion. Unlike mechanisms such as conformational masking, antigenic drift, and glycan shielding, which primarily alter epitope accessibility or viral antigenicity, the bimodal conformation enables the virus to switch between two distinct structural binding modes (Figure 7b). This switching optimizes receptor engagement in a high-affinity state while briefly adopting a low-affinity conformation to reduce neutralizing antibody binding. It surpasses the static conformational masking seen in HIV, influenza, and early SARS-CoV-2 variants, where structural rearrangements shield antigenic sites from immune recognition until receptor binding occurs [63,64,65]. In early SARS-CoV-2 variants, the RBD-down state passively conceals epitopes until receptor engagement triggers an RBD-up transition [65,66]. Similarly, antigenic drift in influenza arises from mutations in immunodominant regions, reducing antibody binding affinity [67,68]. Recent studies show SARS-CoV-2 undergoes similar evolution, with mutations altering neutralizing epitopes and glycosylation patterns to evade antibodies [44,69].

The bimodal strategy provides a dynamic, reversible mechanism, allowing the virus to alternate between high- and low-affinity RBD states, balancing receptor binding with epitope concealment. This complements mutation-driven antigenic drift and refines conformational masking by actively modulating epitope exposure. It leverages structural plasticity, similar to the “cryptic epitope exposure” observed in viral fusion proteins, where transient conformational states allow receptor binding while limiting sustained antibody recognition [70,71]. Hence, the bimodal conformation strategy offers a novel immune escape mechanism in SARS-CoV-2, enhancing viral adaptability. Moreover, as our MD simulations did not account for glycosylation, the potential interaction between the bimodal conformation strategy and glycan shielding is uncertain. Future studies incorporating glycan dynamics are vital to understanding their combined role in immune evasion. Integrating antibody neutralization assays, computational epitope mapping, and structural analyses could further elucidate the impact of conformational flexibility on immune recognition, aiding the development of vaccines and therapeutics against emerging SARS-CoV-2 variants.

Our results have significant implications for vaccine design and therapeutic development. The reduced binding affinity of later Omicron variants to hACE2, coupled with their structural adaptations for immune evasion, highlights the need for next-generation vaccines that address antigenic shifts. Although the structural stability and dynamic properties of the RBD variants make for a good target for small-molecule inhibitors that can disrupt critical binding interactions with hACE2, the frequency of mutations at RBD may hinder their efficacies. Therefore, targeting conserved regions like the S2 domain (Figure S10), which is less prone to mutations and capable of cross-neutralizing multiple coronaviruses, may provide broader efficacy against emerging variants [72]. Antibodies targeting the S2 domain have been found to cross-neutralize multiple coronaviruses [73,74]. As SARS-CoV-2 continues to evolve, integrating computational predictions with experimental data is crucial for designing effective vaccines and therapies to counter future variants.

## 3. Materials and Methods

### 3.1. hACE2–RBD Complex Structure Modeling

From the ancestral WT strain, the first Omicron variant introduces a total of 15 substitutions (G339D, S371L, S373P, S375F, K417N, N440K, G446S, S477N, T478K, E484A, Q493R, G496S, Q498R, N501Y, and Y505H) in the RBD of the ancestral spike protein [75]. To analyze the hACE2–RBD complex structures of various Omicron subvariants to assess the impacts of these mutations and additional substitutions, we retrieved high-resolution crystal structures of the hACE2–RBD complexes for Omicron variants BA.1 (PDB ID: 7WBP) [11], BA.1.1 (PDB ID: 7XAZ) [43], BA.2 (PDB ID: 7XB0) [43] and BA.3 (PDB ID: 7XB1) [43]. Additionally, we included structures of the Delta variant (PDB ID: 7WBQ) [11] and the ancestral WT strain (PDB ID: 6M0J) [76]. For variants that lacked available experimental structures at the time of our research, specifically BA.4/BA.5, XBB.1.9.1, XBB.1.16, BF.7, and JN.1, we constructed homology models using the SWISS-MODEL interactive server (SIB Swiss Institute of Bioinformatics, Basel, Switzerland) [77]. The variant sequences were aligned with the WT template, and 3D structures were generated through template-based modeling.

To validate the stereochemical quality of modeled structures, Ramachandran plots generated by the SWISS-MODEL tool were used. This assessment involved evaluating per-residue geometry and overall structural parameters to confirm that the models are suitable for subsequent molecular dynamics simulations and interaction analyses (Figure S1).

### 3.2. Molecular Dynamics Simulations of hACE2–RBD Complexes

MD simulations were performed to explore the dynamics of the hACE2–RBD complexes for each variant. The complexes were prepared using the CHARMM-GUI webserver [78]. All systems were parametrized with CHARMM36 force fields [79,80] and simulations were performed using GROMACS 2022.1 software [81]. The complexes were solved in a rectangular water box with explicit TIP3P water molecules, maintaining a minimum distance of 1 nm (10 Å) from any protein atom to the box boundaries. Sodium and chloride ions were added to neutralize the systems and maintain a physiological concentration of 0.15 M.

Each system underwent a two-step equilibration process. First, energy minimization was performed using the steepest descent method to remove steric clashes. Subsequently, the system was equilibrated under constant volume (NVT) and constant pressure (NPT) conditions for 1 ns and 3 ns, respectively. The temperature was maintained at 310 K using the V-rescale thermostat [82] and pressure was regulated at 1 bar using the Parrinello-Rahman barostat [83]. During system equilibration, positional restraints were applied on non-hydrogen atoms of hACE2–RBD complexes to ensure stability while allowing the solvent and ions to equilibrate.

Following equilibration, 500 ns production simulations were conducted under NPT conditions. To ensure statistical reliability, the simulations were run in duplicate. The visualizations and analyses of simulation trajectories were performed using Visual Molecular Dynamics (VMD) [84] and UCSF Chimera [85]. In analyzing the trajectories, contacts between hACE2 and RBD were defined as when any two atoms from the interacting residues were within 3.5 Å of each other. Hydrogen bonds were identified based on a donor–acceptor distance cutoff of 3.5 Å and a hydrogen-donor–acceptor angle cutoff of 40 degrees. The buried surface area was calculated by:

(1) $$S_{buried}=S_{hACE2}+S_{RBD}-S_{Complex}$$

where $S_{hACE2}$ and $S_{RBD}$ are the solvent-accessible surface area (SASA) values for the hACE2 receptor and RBD alone, respectively, and $S_{Complex}$ is the SASA of the hACE2–RBD complex.

### 3.3. MM/GBSA Interaction Energy Calculations

For all hACE2–RBD complexes, interaction energy calculations were performed using the Prime MM/GBSA module in Schrödinger suite 2022–4 [86]. To minimize uncertainties arising from dynamic conformational variations, clustering analysis was applied to the MD simulation trajectories of each complex. The largest class represents the conformation that occurs most frequently in the simulation, and its corresponding state is often the most stable state, which can be used as a representative structure. Thus, K-means clustering was employed, and the center of the largest cluster was selected as the representative structure for subsequent MM/GBSA calculations. Prime MM/GBSA uses the OPLS-AA force field and VSGB 2.0 implicit solvation model to estimate the binding energy of the receptor–ligand complex. The binding energy was calculated as:

(2) $$\Delta G\;\left(bind\right)=E_{Complex}-\left(E_{RBD}+E_{hACE2}\right)$$

### 3.4. PMF-Based Binding Free Energy Calculations

To quantitatively evaluate the binding affinities between the SARS-CoV-2 RBD and hACE2 receptor, the PMF approach was applied. The center-of-mass (COM) distance between the RBD and hACE2 was chosen as the reaction coordinate, calculated using the Cα atoms of both proteins. US was utilized to enhance sampling by applying a harmonic biasing potential along the reaction coordinate, facilitating transitions across energy barriers [87]. Initial configurations for US were generated through steered molecular dynamics, where the RBD was pulled incrementally from hACE2 along the reaction coordinate with a pulling velocity of 0.1 nm/ps and a harmonic spring constant of 1000 kJ/mol/nm^2^. Snapshots were extracted at 0.2 nm intervals along the reaction coordinate, extending up to 4.5 nm from the bound state. Each snapshot served as a starting structure for an umbrella sampling window. The simulations were conducted with harmonic restraints, defined as:

(3) $$U_{us}\left(R_{i},R\right)=\frac{1}{2}k{\left(R_{i}-R_{hACE2-RBD}\right)}^{2}$$

where *k* is the force constant, set to 10 kcal/(mol·Å^2^) to ensure sufficient overlap between adjacent windows. *R_hACE2–RBD_* is the biased distance between the COM of RBD and hACE2 $R_{i}$ is the restraint position for each window.

Each window was equilibrated and simulated for 2–5 ns to ensure adequate sampling. The free energy profile was reconstructed using the Weighted Histogram Analysis Method (WHAM), combining data across all windows to obtain the PMF as a function of the COM distance. The global minimum of the PMF represents the bound state free energy, while the plateau at large COM distances indicates the unbound state. The binding affinity was calculated as the difference between these two states, representing the relative binding free energy of the hACE2–RBD complex.

(4) $${∆G}_{bind}={PMF}_{unbound}-{PMF}_{bound}$$

PMF convergence was validated by monitoring histogram overlaps between neighboring windows and ensuring consistency across replicates.

### 3.5. Principal Component and Free Energy Landscape Analysis

To investigate the dominant motions and dynamic conformational changes of the proteins over time, PCA was performed on each trajectory. The MD Analysis library [88] in Python 3.7.3 was utilized to process and analyze the MD trajectories, while the scikit-learn library [89] was used to conduct the PCA. Additionally, FEL was constructed for all variants to gain insights into their stability and functional behavior. The FEL was calculated using the following formula:

(5) $$∆G\left(variants\right)=-Tk_{B}lnP(variants)$$

where the $k_{B}$ is the Boltzmann constant and the *T* is the absolute temperature. $P\left(variants\right)$ represents the probability distribution of the molecular system states for a given variant. Prior to analysis, each frame of the trajectory was aligned to a reference structure to eliminate global translational and rotational movements.

## 4. Conclusions

This study underscores the role of mutations in modulating the interaction between the SARS-CoV-2 RBD and the hACE2 receptor. Binding affinity as assessed through PMF and MM/GBSA calculations demonstrates substantial variability across variants. This variability, when compared to the experimental *K*_D_ values, shows that enhanced receptor binding is not uniformly favored during viral evolution. Earlier variants, such as Delta and BA.1, show strong receptor interactions, while later Omicron subvariants, such as JN.1, have moderate binding strength and exhibit a bimodal conformational strategy. This may reflect a shift toward balancing moderate receptor binding with robust immune escape mechanisms.

These mutations significantly influence the structural stability of the viral RBD and the strength of its interactions with hACE2. Notably, we observed that the early Omicron RBD variants exhibit higher positive charges at the interface than the WT, enhancing electrostatic interactions with hACE2. Additionally, mutations in Omicron variants contribute to greater structural stability, as reflected by reduced RMSD values, and this resulted in an increased number of contacts with hACE2 relative to WT.

These findings suggest that the evolutionary trajectory of SARS-CoV-2 is shaped by a multifaceted interplay between structural stability, binding affinity, and potentially other factors, such as immune evasion, transmissibility, or replication efficiency. Hence, SARS-CoV-2 may be “fine-tuning” interactions to evade host immunity while maintaining sufficient receptor engagement for efficient infection. Such trade-offs are evident in the diverse binding strengths and structural adaptations observed among Omicron sub-lineages.

These findings emphasize the value of integrating complementary computational approaches, such as MD simulation, MM/GBSA, and PMF, to provide robust insights into binding energetics. Moreover, our results highlight the importance of the continued surveillance of SARS-CoV-2 variants, coupled with adaptive strategies for vaccine design and therapeutic development, to effectively address the ongoing evolution of the virus. Future studies combining advanced computational techniques with experimental validation will be critical for resolving discrepancies.

## Figures and Tables

**Table 1:** Summary of interaction metrics between variants RBD and hACE2 during the final 100 ns of the equilibrium simulation.

Variants	Number of H-Bonds	Number of Contacts	Buried Surface Area (nm^2^)	*K*_D_ Values (nM) *	References
Wild Type	6.02	263.39	17.73	24.4, 21.9, 16.4	[43,44,45]
Delta	7.57	316.28	18.42	25.1, 13.5, 2.85	[43,44,46]
BA.1	9.99	276.97	18.17	19.5, 14.5, 9.74, 8.85	[43,44,45,46]
BA.1.1	5.49	293.07	18.31	5.9	[43]
BA.2	8.24	312.61	18.98	10.0, 10.8, 2.99	[43,44,45]
BA.3	9.22	313.23	19.10	22.1, 26.5	[43,44]
BA.4/5	4.97	274.95	17.51	14.4, 3.1, 10.7	[44,45,47]
BF.7	4.57	263.06	17.59	12.97	[47]
XBB.1.16	7.38	285.76	18.53	3.2	[45]
XBB.1.9.1	5.35	286.20	17.89	3.5, 13.90, 6.0	[45,47,48]
JN.1	7.00	283.58	19.37	13	[49]

* The *K*_D_ value was determined by Surface Plasmon Resonance (SPR).

## Data Availability

The data used to support the findings of this study are available from the co-first authors upon reasonable request.

## Supplementary Materials

The following supporting information can be downloaded at: https://www.mdpi.com/article/10.3390/ijms26083776/s1.
