# Supplementary material for: Structural and Energetic Insights into SARS-CoV-2 Evolution: Analysis of hACE2–RBD Binding in Wild-Type, Delta, and Omicron Subvariants
Source: Int J Mol Sci. 2025 Apr 17;26(8):3776. doi: 10.3390/ijms26083776 (PMC12027596; doi:10.3390/ijms26083776)
Supplement: Supplementary file 1 [file ijms-26-03776-s001.zip › ijms-3517301-supplementary.pdf]

Supplementary information for:

Structural and energetic insights into SARS-CoV-2 evolution: Analysis of hACE2-RBD binding in Wild-Type, Delta, and Omicron Subvariants

Can Tang[1,2,#], Cecylia S. Lupala[1,#,*], Ding Wang[3], Xiangcheng Li[4,5], Lei-Han Tang[6,*] and Xuefei Li[1,*]

[1] *Key Laboratory of Quantitative Synthetic Biology, Shenzhen Institute of Synthetic Biology, Shenzhen Institutes of Advanced Technology, Chinese Academy of Sciences, Shenzhen 518055, China*

[2] *University of Chinese Academy of Sciences, Beijing 100049, China*

[3] *Department of Physics, Hong Kong Baptist University, Hong Kong SAR, China*

[4] *School of Life Science and Technology, ShanghaiTech University, Shanghai, China.*

[5] *Shanghai Institute for Advanced Immunochemical Studies, ShanghaiTech University, Shanghai, China.*

[6] *Center for Interdisciplinary Studies, Westlake University, Hangzhou 310024, China*

Table S1: Variants grouping based on MM/GBSA and PMF results

Category	*Variants*	*PMF (kcal/mol)*	*MM/GBSA (kcal/mol)*
Strong Binders *(PMF<-30 or MMGBSA<-110)*	Delta, BA.1, BA.3	-31.25, -27.7, -21.9	-107.344, -133.515, -110.689
Moderate Binders *(-30<PMF<-20 or -110<MM/GBSA<-70)*	BA.1.1, BA.2, BA.4/5, BF.7, XBB.1.16, XBB.1.9.1, JN.1	-21.8, -21.7, -26.6, -29.25, -24.7, -23.9, -28.2	-96.338, -103.511, -81.868, -80.828, -108.318, -91.25, -54.19
Weak Binders *(PMF>-20 and MM/GBSA>-70)*	WT	-11.85	-64.281

Table S2: Variants classification based on MD simulation metrics

Group	*Variants*	*Rationale (MD descriptors)*
Strong binders *(#H-bonds ≥8, Contacts ≥310 BSA ≥19.0 nm^2)*	BA.3, BA.2, Delta	High H-bonds, contacts, and/or BSA.
Moderate binders *(#H-bonds 6-8, Contacts 280-310 BSA 18.0-19.0 nm^2)*	XBB.1.16, BA.1.1, JN.1, BA.1	Intermediate values for H-bonds and contacts.
Weak binders *(H-bonds <6, Contacts <280 BSA<18.0nm^2)*	WT, BF.7, BA.4/5, XBB.1.9.1	Low H-bonds, contacts, and smaller BSA.

Table S3: Variants classification based on reported K_D values range

Group	*Variants*	*K_D (nM)*
Strong Binders	Delta, BA.2, XBB.1.16	<5 nM
Moderate Binders	BA.1.1, BA.1, BA.4/5, JN.1, XBB.1.9.1, BF.7	5nM−15nM
Weak Binders	WT, BA.3	≥15nM

Table S4: MD interactions metrics and reported experimental K_D values.

Variant	*Year*	*MD Classification*	*K_D Classification*
**Wild Type**	2019	Weak Binder	Weak Binder
**Delta**	2020	Strong Binder	Strong Binder
**BA.1**	2021	Moderate Binder	Moderate Binder
**BA.1.1**	2021	Moderate Binder	Moderate Binder
**BA.2**	2021	Strong Binder	Strong Binder
**BA.3**	2022	Strong Binder	Strong Binder
**BA.4/5**	2022	Weak Binder	Weak Binder
**BF.7**	2022	Weak Binder	Weak Binder
**XBB.1.16**	2023	Moderate Binder	Moderate Binder
**XBB.1.9.1**	2023	Weak Binder	Moderate Binder
**JN.1**	2023	Moderate Binder	Moderate Binder

Table S5: The eigenvalues and variance ratio for the first two principal components of the variants RBD.

Variants	*PC1 Eigenvalue*	*PC2 Eigenvalue*	*PC1 Variance Ratio*	*PC2 Variance Ratio*
**WT**	69.01	34.48	0.30	0.15
**Delta**	56.04	41.87	0.22	0.17
**BA.1**	55.49	44.32	0.22	0.17
**BA.2**	47.35	35.08	0.19	0.14
**BA.3**	103.11	40.22	0.35	0.14
**BA.4/5**	61.25	46.51	0.23	0.18
**BA.1.1**	59.25	38.06	0.22	0.14
**BF.7**	53.46	40.34	0.20	0.15
**XBB.1.9.1**	62.16	32.86	0.24	0.13
**XBB.1.16**	80.31	34.25	0.31	0.13
**JN.1**	90.15	43.80	0.33	0.16

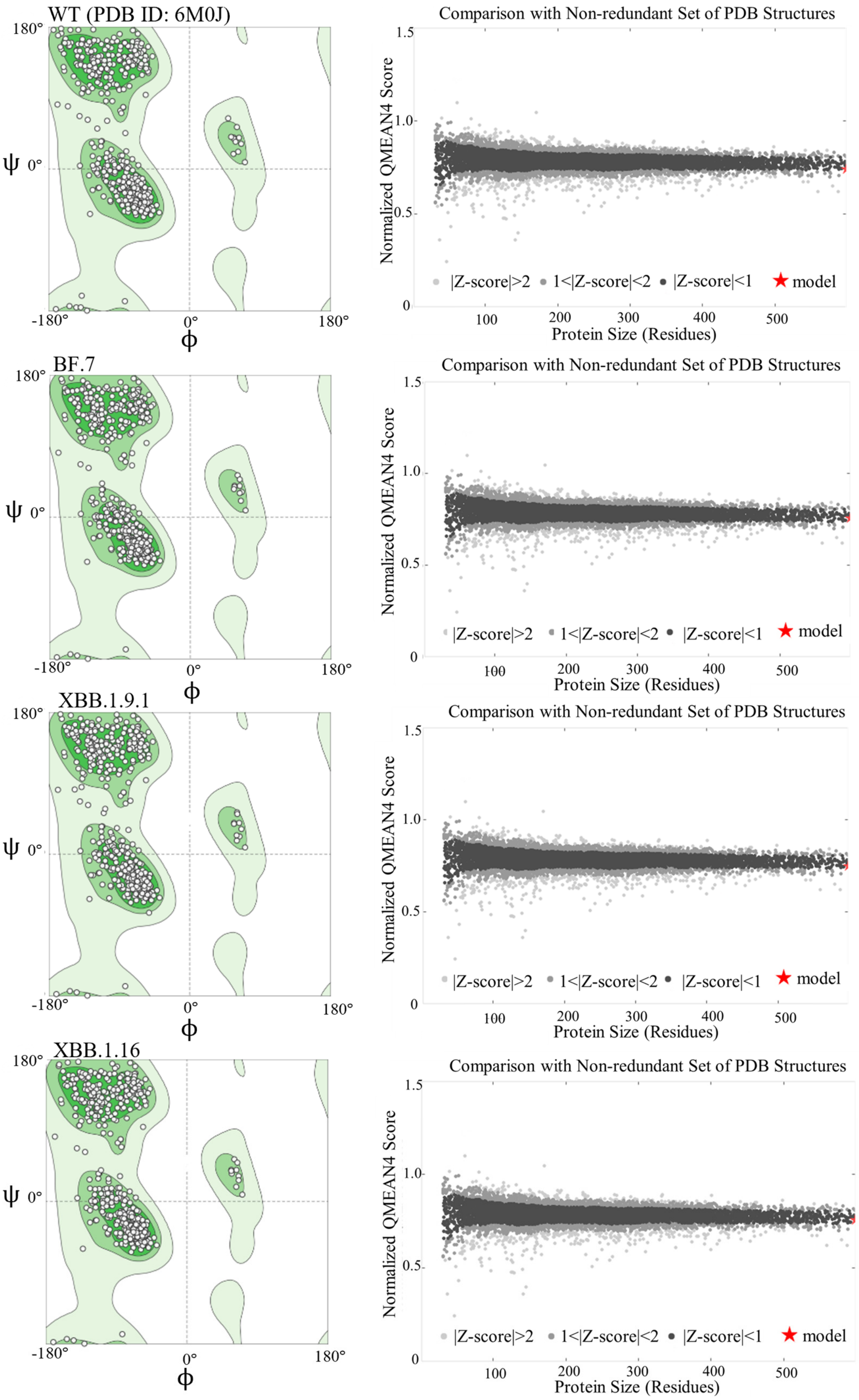

Figure S1. Model assessment and validation. Comparison of quality metrics for the predicted structures of the RBD-hACE2 complex (BF.7, XBB.1.9.1, XBB.1.16) and the WT complex, supported by Ramachandran plots, confirms that all predicted structures exhibit good quality.

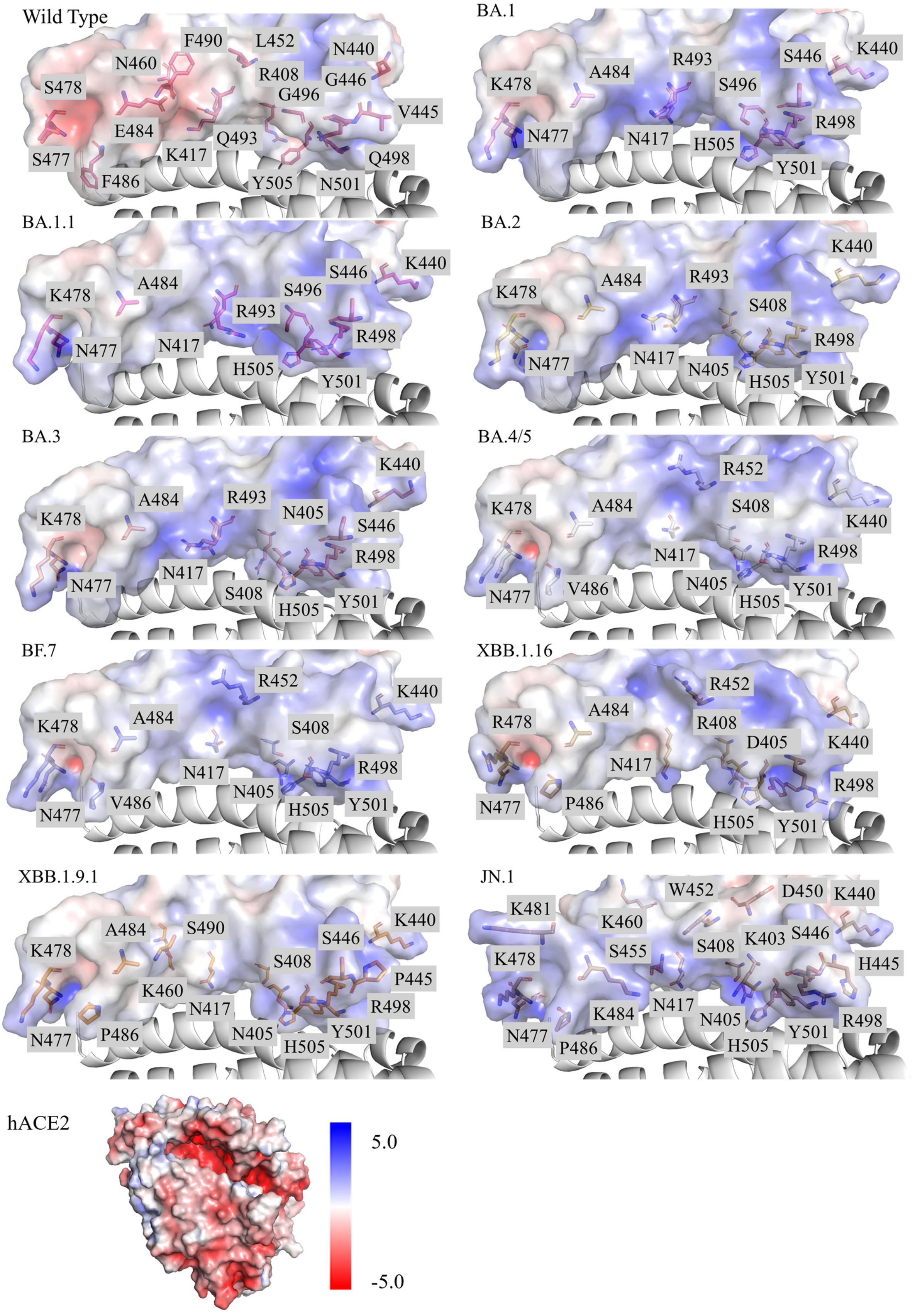

Figure S2. Electrostatic surface potentials of RBD variants bound to hACE2

Electrostatic surface potentials of RBD variants bound to hACE2 are shown, with a gradient ranging from -5 k_BT/e (red, negative potential) to +5 k_BT/e (blue, positive potential). Mutation sites at the interface are shown as sticks, and hACE2 is depicted as a white cartoon for clarity. An increase in positive electrostatic potential at the hACE2-RBD interface is evident across SARS-CoV-2 evolution, reflecting a better charge complementarity to the more negatively charged hACE2 surface.

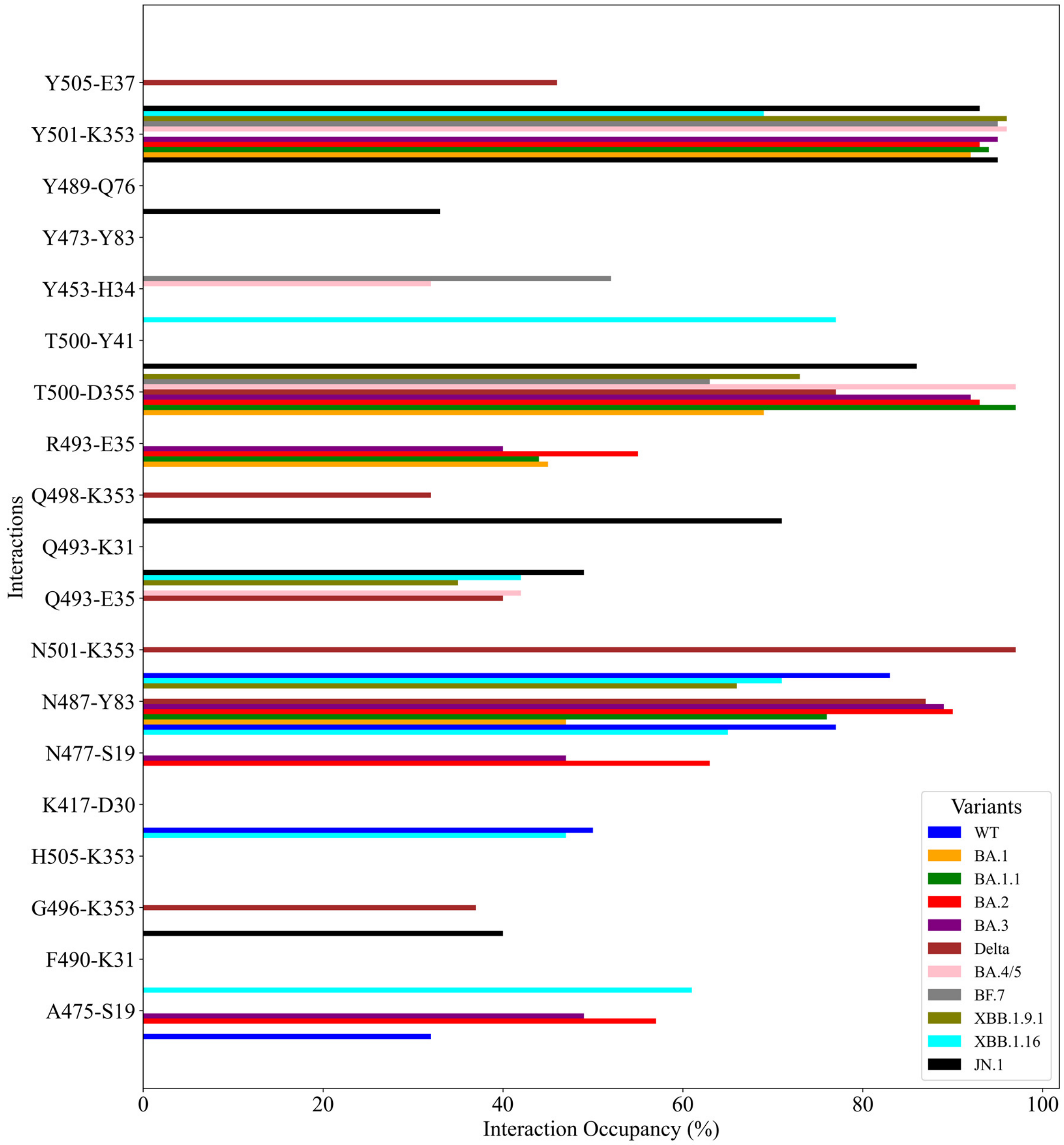

Figure S3. Hydrogen bond interaction occupancy across variants. The horizontal bar chart shows the interaction occupancy of various interactions across multiple variants based on the results of the 500 ns equilibrium simulation. Only interactions with at least one occupancy value greater than or equal to 30% are included in the chart. Some mutations, such

as N501Y and Q493R, show a higher H-bond occupancy.

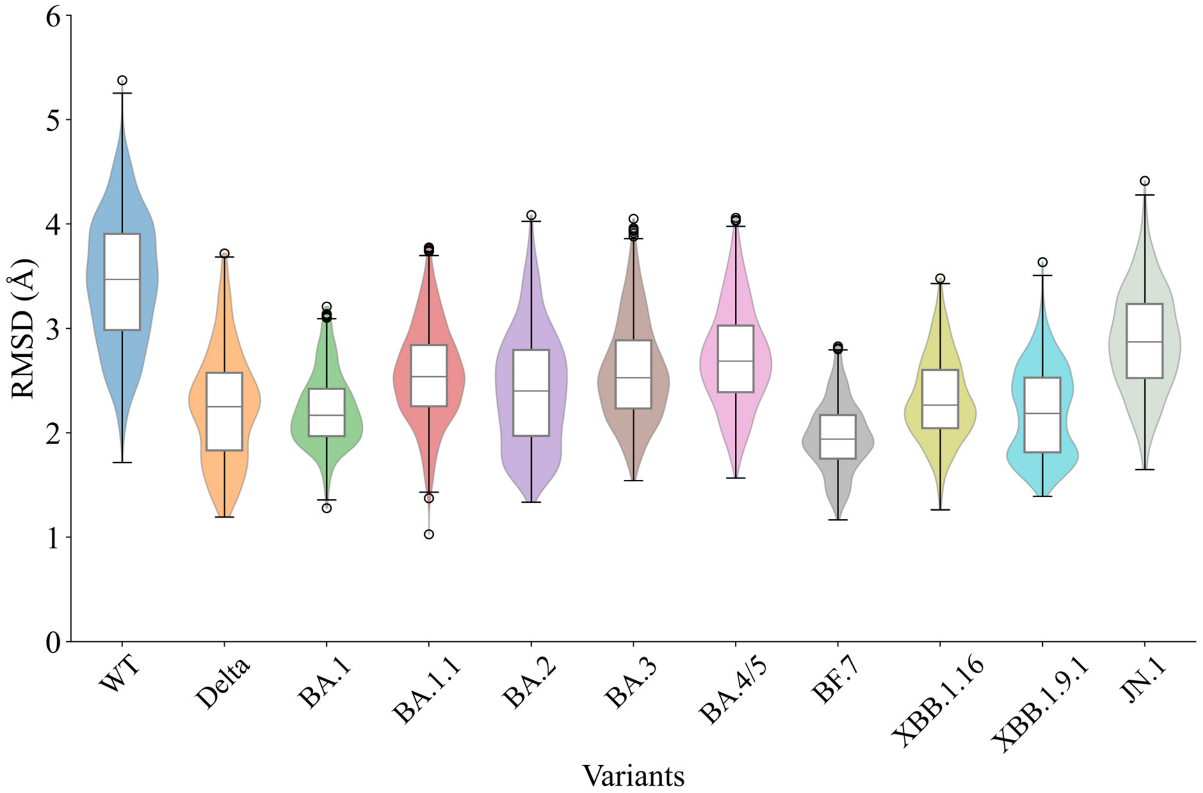

Figure S4. The RMSD of the backbone atoms of the RBD-hACE2 complexes with respect to the starting structure for two trajectories. WT shows the highest value relative to Omicron and Delta, reflecting enhanced structural stability during evolution.

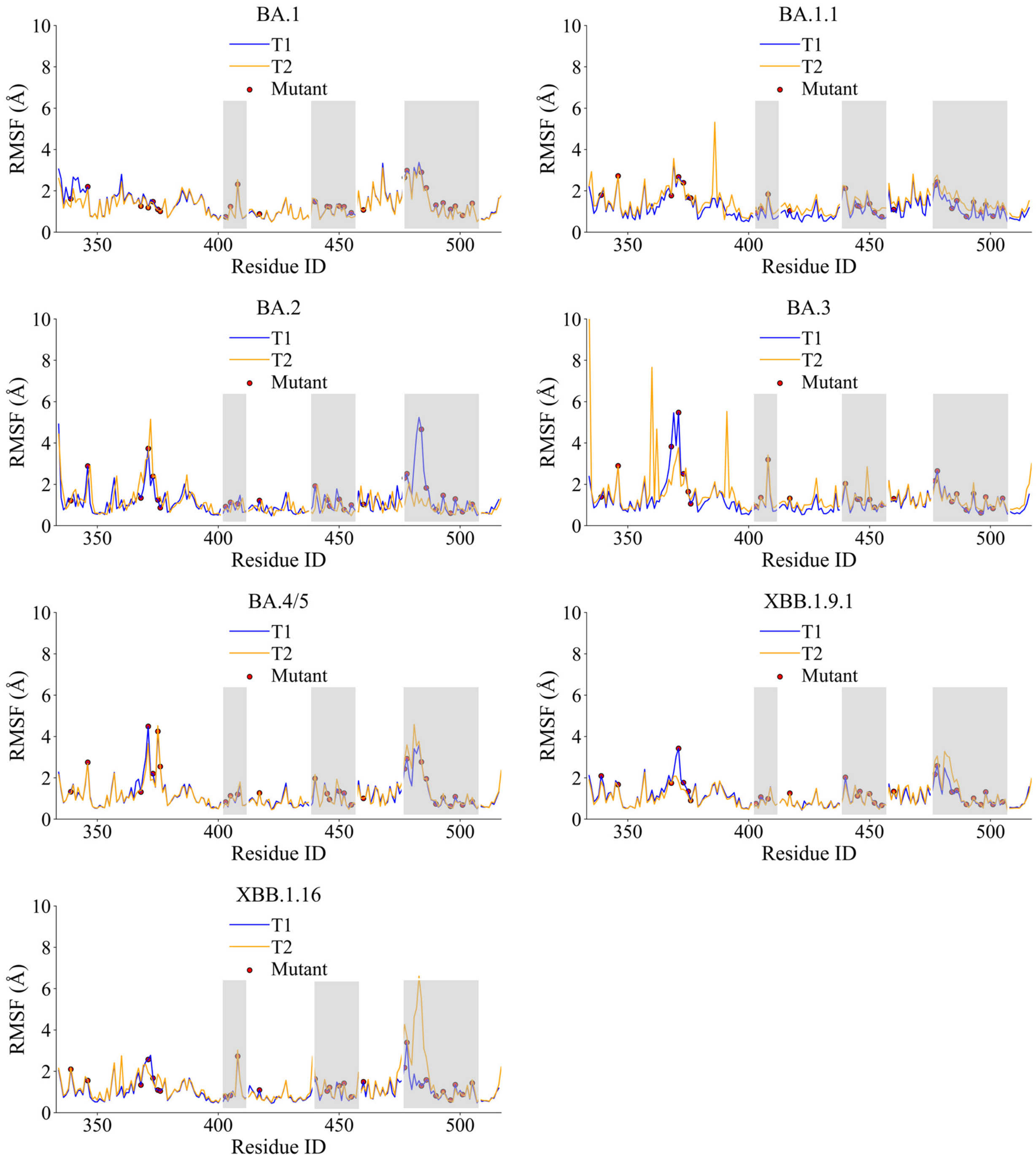

Figure S5. The RMSF profiles for the variants' RBD residues during the two independent 500 ns MD simulations. T1 and T2 represent the first and second simulations. Mutation residues are also labeled, and the interfaces are highlighted with shadow. Most variants exhibit high RMSF values at the loop tip residues, indicating greater flexibility in these regions. In comparison, BA.3 and XBB.1.16 show increased flexibility, with higher RMSF values across a broader range of residues.

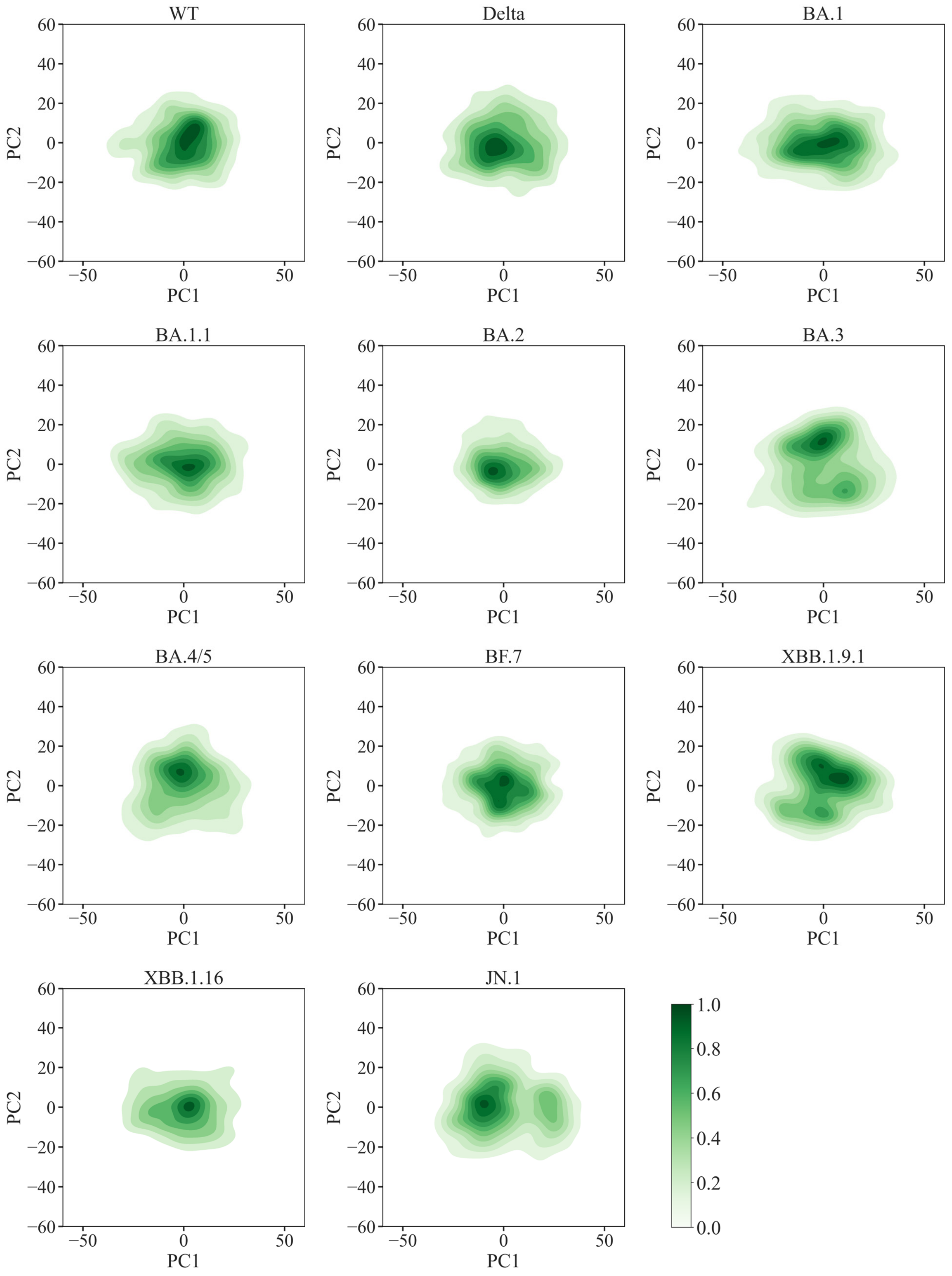

Figure S6: The projection of the hACE2-RBD complex trajectory. Most variants exhibit a single dense conformational region, indicating conformational stability, while BA.3 and JN.1 display distinct conformational subpopulations, suggesting possible variations in binding

behavior.

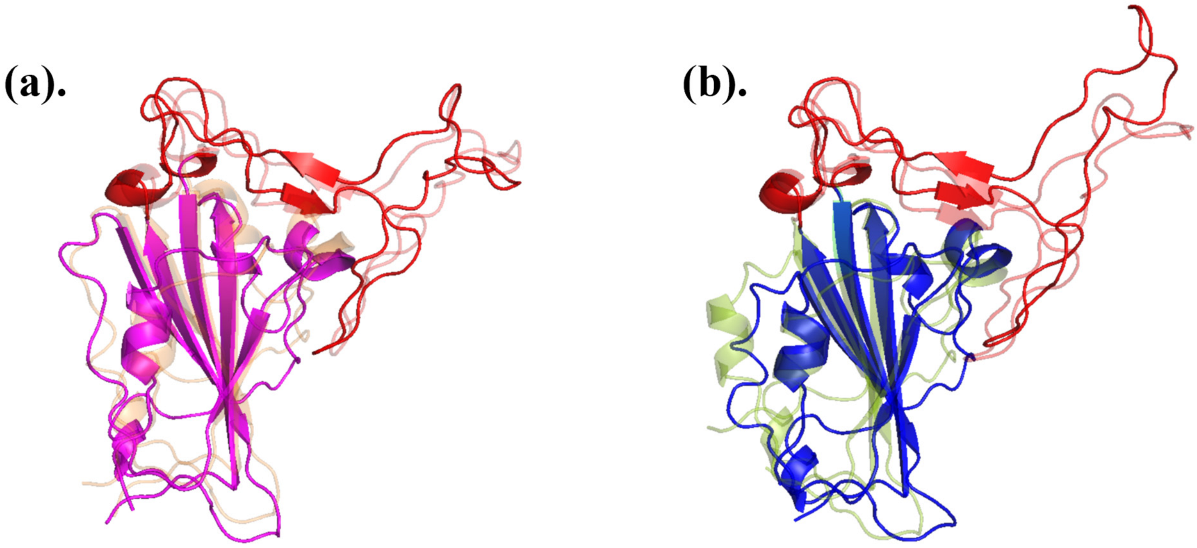

Figure S7. Structure mapping of the two conformations of BA.3 and JN.1 (a)In BA.3, and (b) in JN.1, the RBM structures are highlighted in red. The differences between the multiple conformations are mostly on the loop at the tip of RBD and patch *(iii)* of the interface.

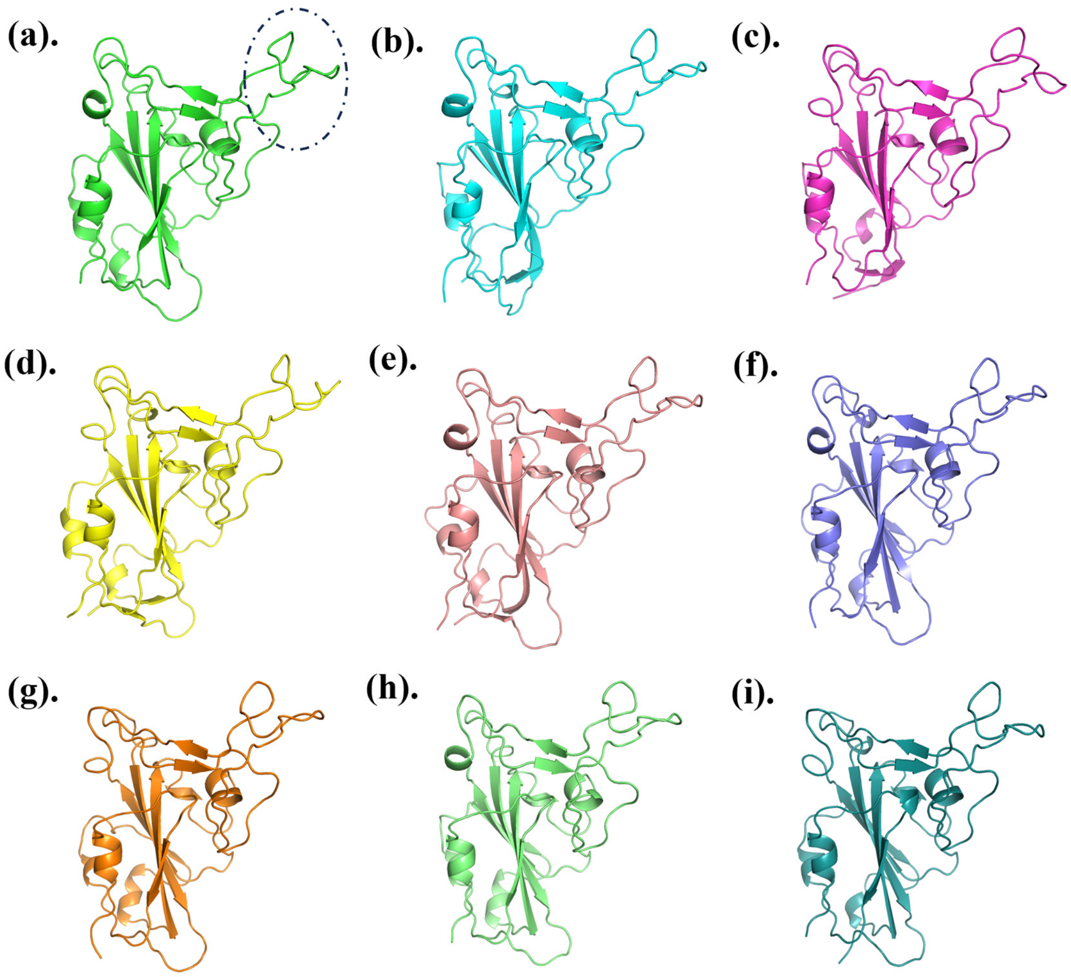

Figure S8. Representative structures from largest cluster center identified in the PCA analysis. (a) WT, (b) Delta, (c) BA.1, (d) BA.1.1, (e) BA.2, (f) BA.3, (g) BA.4/5, (h)XBB.1.16, and (i)XBB.1.9.1. The "hook-like" architectures are highlighted with circles, and most variants retained this architecture at the interface essential for hACE2 binding.

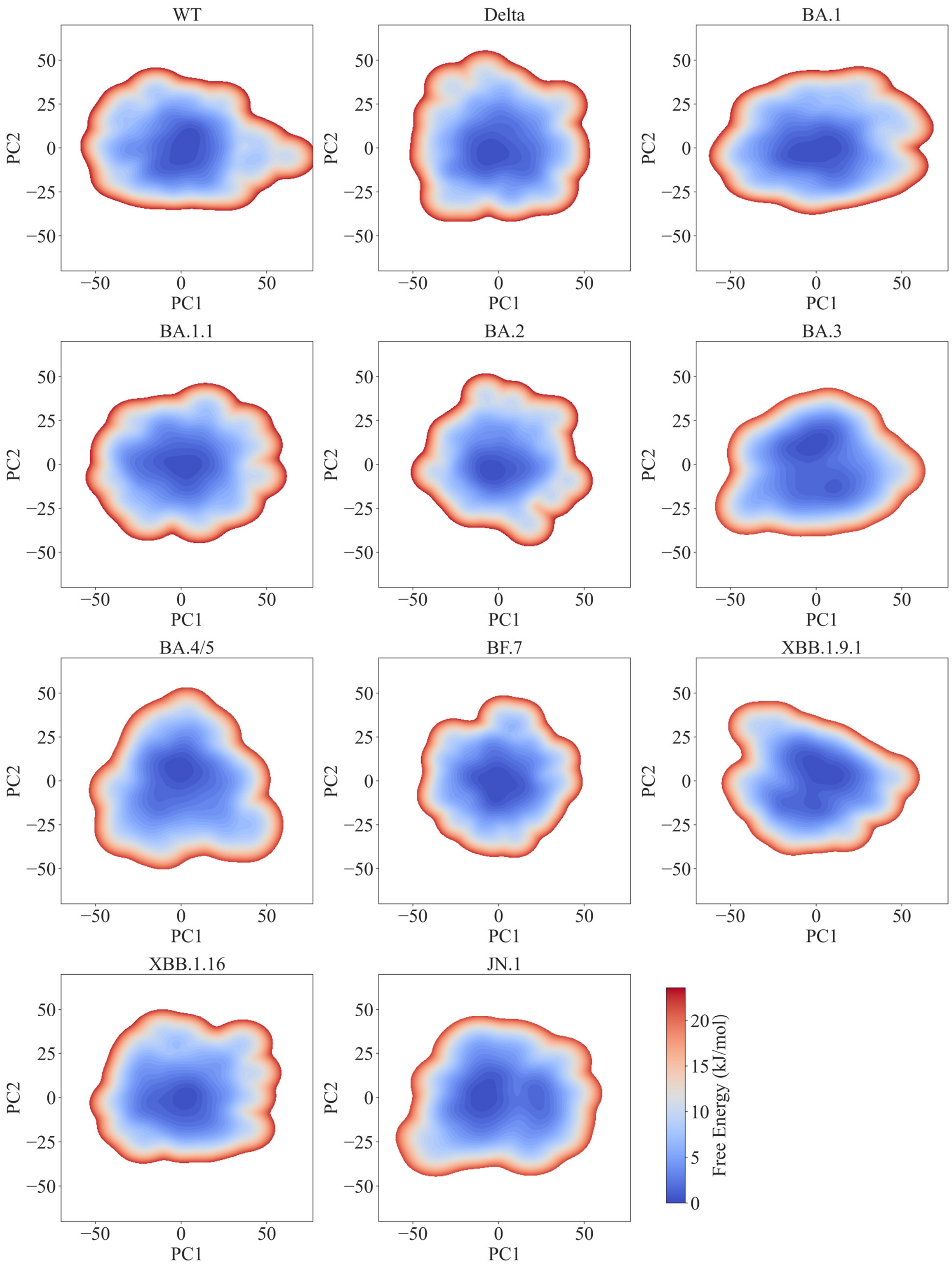

Figure S9. The free energy landscape of hACE2-RBD complexes based on PCA. FEL is made from 500 ns MD simulations with the interval of 1 ns. Most variants like WT, Delta, and BF.7 show centralized, symmetrical basins while JN.1 and BA.3 showed broader and fragmented landscapes.

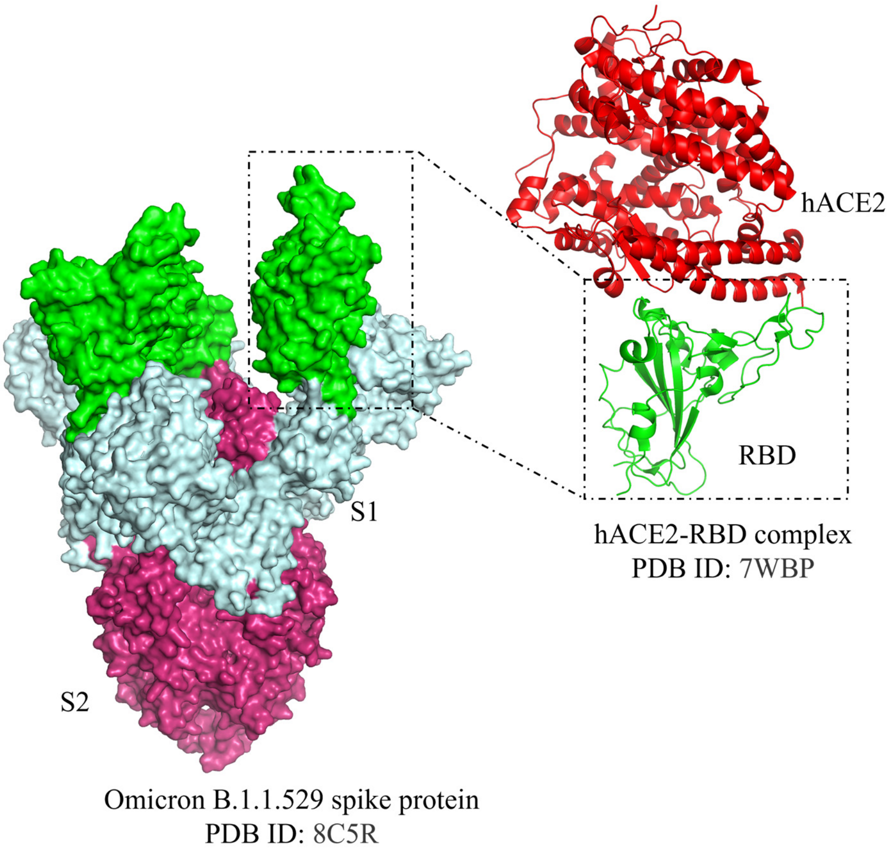

Figure S10. Full-length structure of the SARS-CoV-2 spike protein. The spike protein is a trimeric complex formed by three identical chains. The S2 domain, shown in magenta, represents a highly conserved region critical for membrane fusion, exhibiting fewer mutations across variants. The RBD, depicted in green, is a frequently mutated region within the S1 subunit (cyan), key to binding the hACE2, plotted in red, illustrating the RBD-hACE2 interaction.
